# Homozygous Familial Hypercholesterolemia in a Seven-Year-Old: A Case Study Highlighting the Importance of Early Diagnosis

**DOI:** 10.7759/cureus.86219

**Published:** 2025-06-17

**Authors:** Dalal Bensabbahia, Meriem El Achiwi, Meriem Atrassi, Abdelhak Abkari, Gueddari Widad

**Affiliations:** 1 Department of Pediatrics 3, Pediatric Gastroenterology and Hepatology Unit, Abderrahim Harouchi Mother and Child Hospital, Ibn Rochd University Hospital, Casablanca, MAR; 2 Department of Pediatric Emergency Medicine, Abderrahim Harouchi Mother and Child Hospital, Ibn Rochd University Hospital, Casablanca, MAR

**Keywords:** atherosclerosis, genetic analysis, homozygous familial hypercholesterolemia, statins, xanthoma

## Abstract

Familial hypercholesterolemia (FH) is a common autosomal dominant disorder characterized by markedly elevated low-density lipoprotein (LDL)-cholesterol levels and an increased risk of premature cardiovascular disease. The homozygous form, which is much rarer and more severe, manifests in early childhood with extremely high LDL-cholesterol levels and early-onset atherosclerosis.

We report the case of a seven-year-old girl, born to consanguineous parents, presenting with tuberous and tendinous xanthomas. Her lipid profile revealed severe hypercholesterolemia (total cholesterol: 7.5 g/L; LDL-C: 6.82 g/L), low high-density lipoprotein-cholesterol (HDL-C) (0.29 g/L), and normal triglycerides. Echocardiography showed atheromatous lesions in the aortic arch.

Molecular analysis identified a pathogenic homozygous mutation in the LDLR gene. Treatment with atorvastatin followed by ezetimibe was initiated, along with dietary and lifestyle modifications. Follow-up showed moderate regression of the atheromatous lesions. A six-month follow-up plan was established, and LDL apheresis was considered.

This case highlights the importance of early screening, genetic confirmation, and intensive multidisciplinary management to prevent premature cardiovascular complications in children with homozygous familial hypercholesterolemia.

## Introduction

Familial hypercholesterolemia (FH) is an autosomal dominant genetic disorder characterized by persistently elevated low-density lipoprotein (LDL) cholesterol levels and an increased risk of premature cardiovascular complications [1]. Its homozygous form (HoFH), which is rare and severe, affects approximately one to three individuals per million and manifests in early childhood with severe hypercholesterolemia, early-onset xanthomas, and accelerated atherosclerosis [2,3]. Diagnosis is based on clinical and biological criteria, along with the identification of biallelic pathogenic variants in the LDLR, APOB, or PCSK9 genes [2,4]. Pediatric management combines strict dietary and lifestyle modifications, high-dose statins, ezetimibe, and, in some cases, LDL apheresis starting as early as five years of age [1,3]. This case highlights the need for rigorous multidisciplinary follow-up from the time of diagnosis to optimize therapeutic management and improve long-term cardiovascular outcomes in children with HoFH.

## Case presentation

**Figure 1:**
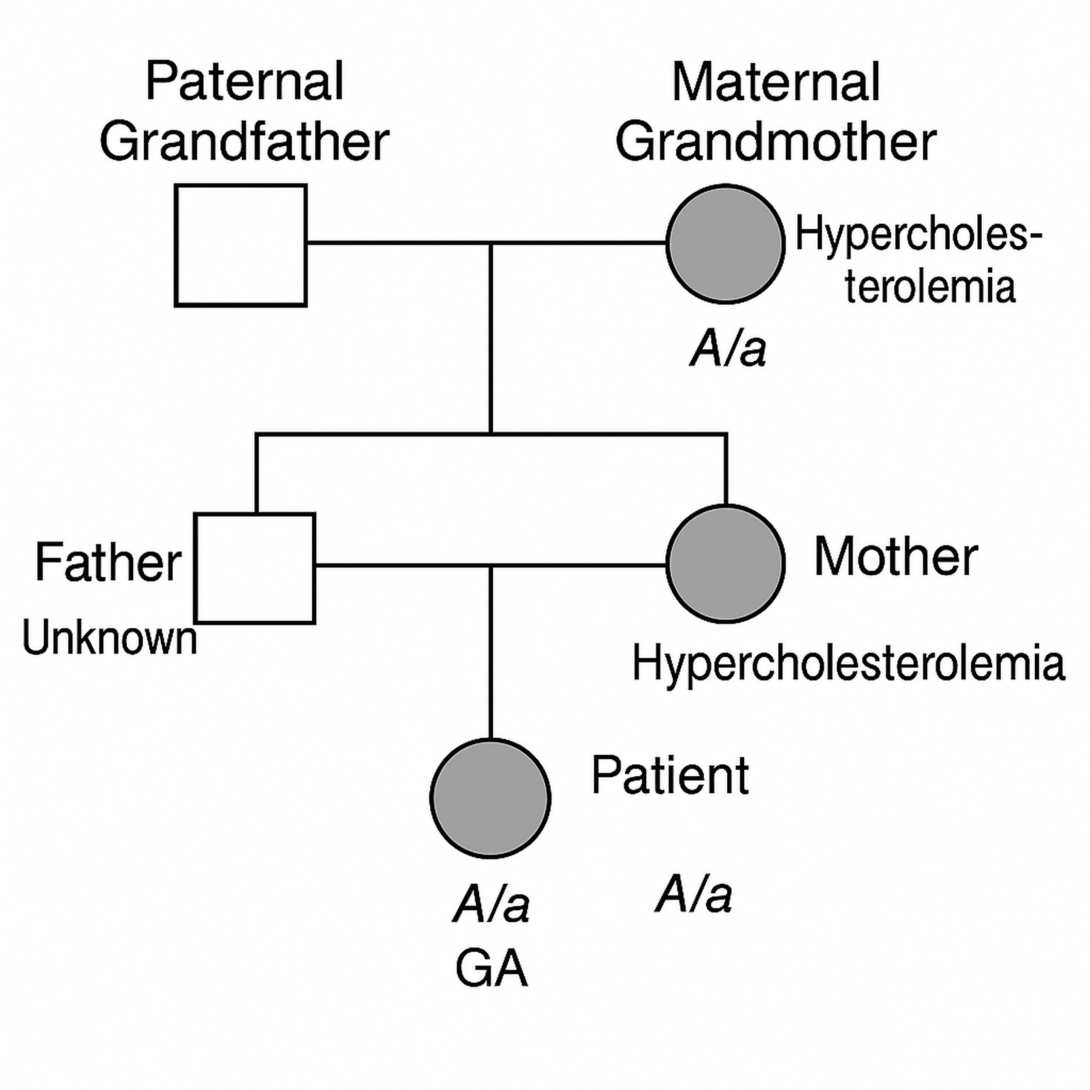
Pedigree diagram to illustrate the case of family history A/a: Heterozygous genotype (one normal and one mutated allele) GA: Genetic analysis performed and confirmed Adapted by the authors from: [5]

We report the case of a seven-year-old girl. She was admitted to our department for an assessment of cutaneous xanthomas. She is the product of a first-degree consanguineous marriage and has a family history of hypercholesterolemia (Figure 1), hypertension in her paternal grandfather, and hypercholesterolemia in her maternal grandmother. The girl had no past medical history or treatment likely to alter lipid metabolism.

On physical exam, she had a weight of 17 kg, corresponding to a deviation of minus two standard deviations for age, while her height of 114 cm was within the norm. Her body mass index (BMI) was 13.1 kg/m², placing her in the lower percentiles for age. Blood pressure was normal (103/56 mmHg). The cardiovascular exam was normal.

**Figure 2:**
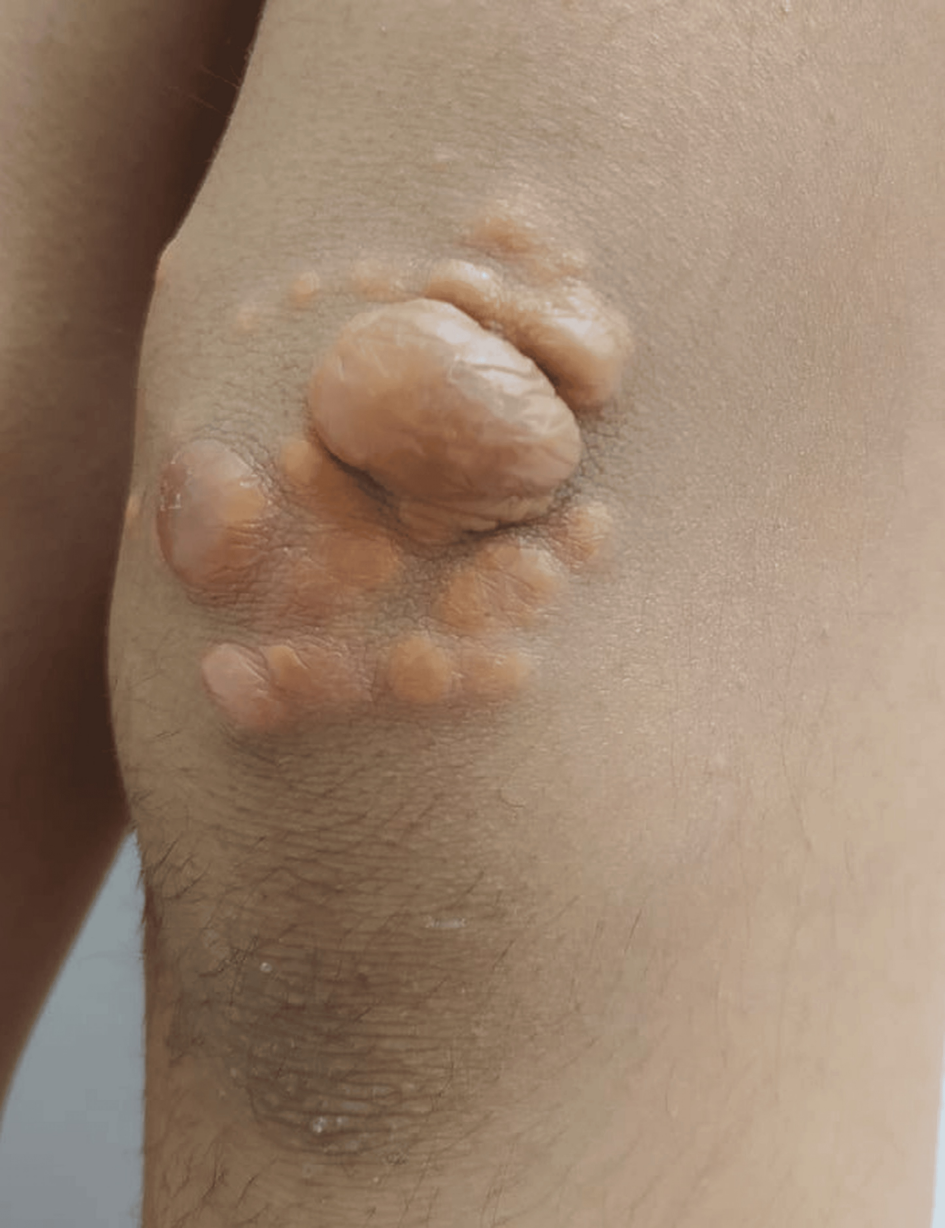
Tendinous xanthomas over the knee

**Figure 3:**
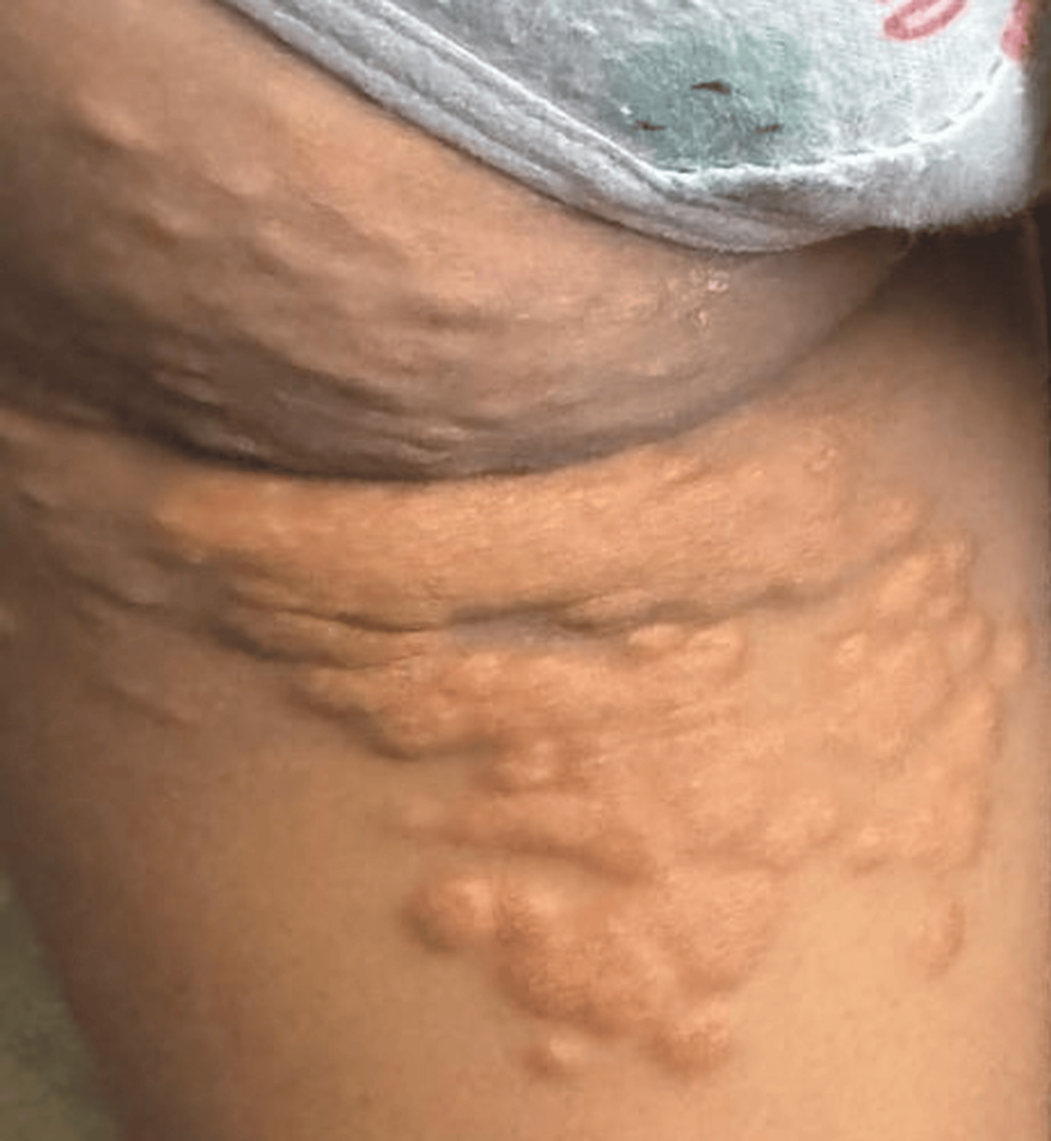
Tuberous xanthomas located on the posterior aspect of the thighs

On skin exam, there were tuberous xanthomas on the posterior aspect of the thighs, and bilateral tendinous xanthomas on the knees and elbows (Figures 2, 3). She had no corneal arches on the ophthalmological exam. These clinical manifestations, together with the family history, led to the diagnosis of suspected severe dyslipidemia of genetic origin.

The lipid profile revealed major hypercholesterolemia, as summarized in Table 1, along with the rest of the lipid profile.

**Table 1:** Lipid profile results of the patient LDL: low-density lipoprotein; HDL: high-density lipoprotein Source of pediatric reference ranges [6]

Parameter	Measured value	Pediatric reference ranges
Total cholesterol	7.5 g/L	< 1.7 g/L (acceptable), 1.7–1.99 g/L (borderline), ≥ 2.0 g/L (high)
LDL cholesterol	6.82 g/L	< 1.1 g/L (acceptable), 1.1–1.29 g/L (borderline), ≥ 1.3 g/L (high)
HDL cholesterol	0.29 g/L	≥ 0.4 g/L (normal), < 0.4 g/L (low)
Triglycerides	1.59 g/L	< 1.1 g/L (ages 0–9), < 1.3 g/L (ages 10–19)

The family lipid panel confirmed hypercholesterolemia in the mother (total cholesterol: 2.80 g/L), maternal grandfather (total cholesterol: 3.28 g/L), and paternal grandmother (total cholesterol: 3.07 g/L).

**Figure 4:**
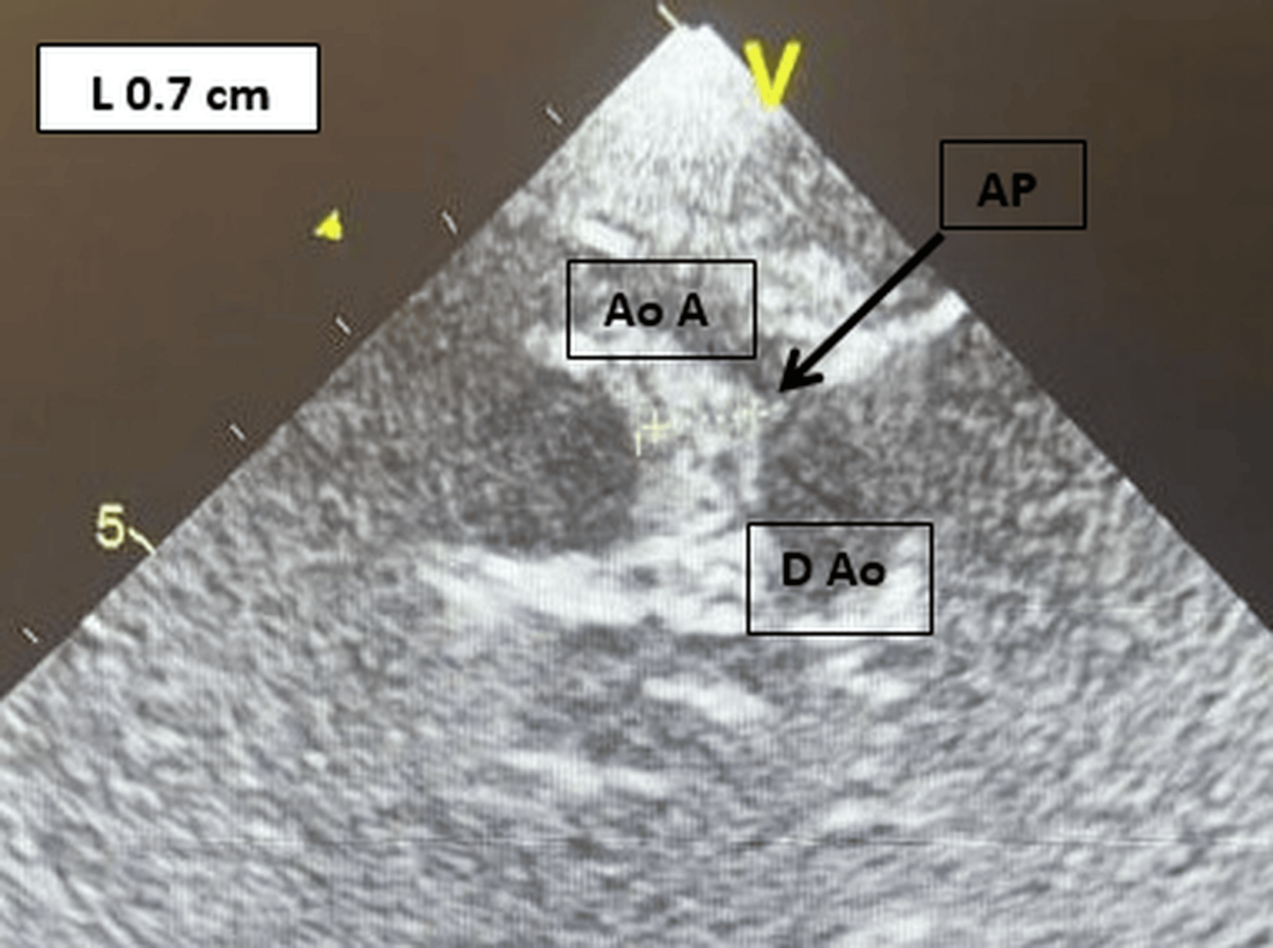
Two-dimensional echocardiographic image from the suprasternal window of large atherosclerosis plaque in the aortic arch. The plaque is 7 mm thick. Ao A is the aortic arch, AP is the atherosclerosis plaque, and D Ao is the descending aorta. The arrow indicates the atherosclerotic plaque in the aortic arch.

Assessment of complications revealed normal liver and kidney function, with no proteinuria. On echocardiography, there was an atheroma in the aortic arch, with no significant stenosis or calcification (Figure 4).

Genetic analysis

Molecular analysis identified a homozygous pathogenic mutation in the LDLR gene, corresponding to variant NM_000527.5: c.682G>C (p.Glu228Gln), which corresponds to SNP rs121908029 in genomic databases, facilitating cross-referencing and variant tracking, associated with familial hypercholesterolemia type 1 (OMIM: 143890). This mutation is extremely rare in the general population, with a frequency of less than 0.001% in the gnomAD database.

To assess its potential pathogenicity, in silico predictive analyses were performed using bioinformatics tools that evaluate the impact of genetic variants based on multiple parameters. These analyses incorporate factors such as evolutionary conservation, structural changes in the protein, and functional disruption of key domains. The mutation received a high deleteriousness score from two major predictive models: REVEL (0.94) and 3Cnet (1.00), both of which suggest a strong likelihood of pathogenicity.

Additionally, the mutation is referenced as pathogenic in clinical databases such as ClinVar (VCV000251393). In our patient, it is located at genomic position 19:11105588G>C (GRCh38), leading to a nucleotide substitution c.682G>C and altering the structure of the encoded LDL receptor protein. This specific region is recognized as a mutational hotspot, meaning it is particularly prone to pathogenic genetic alterations that can impair LDL receptor function, ultimately leading to severe hypercholesterolemia.

Care and follow-up

Treatment was initiated with atorvastatin 10 mg daily, in addition to hygienic dietary measures aimed at limiting animal fat consumption, favoring fatty acids of plant origin, and favoring a diet rich in fiber. 

After six months of follow-up, although a moderate reduction in total cholesterol was observed (6.8 g/L), LDL-cholesterol levels remained high (5.68 g/L). In view of this persistent elevation in LDL-cholesterol and the major cardiovascular risk, an inhibitor of intestinal cholesterol absorption, ezetimibe (10 mg/d), was added to treatment to improve lipid control.

**Figure 5:**
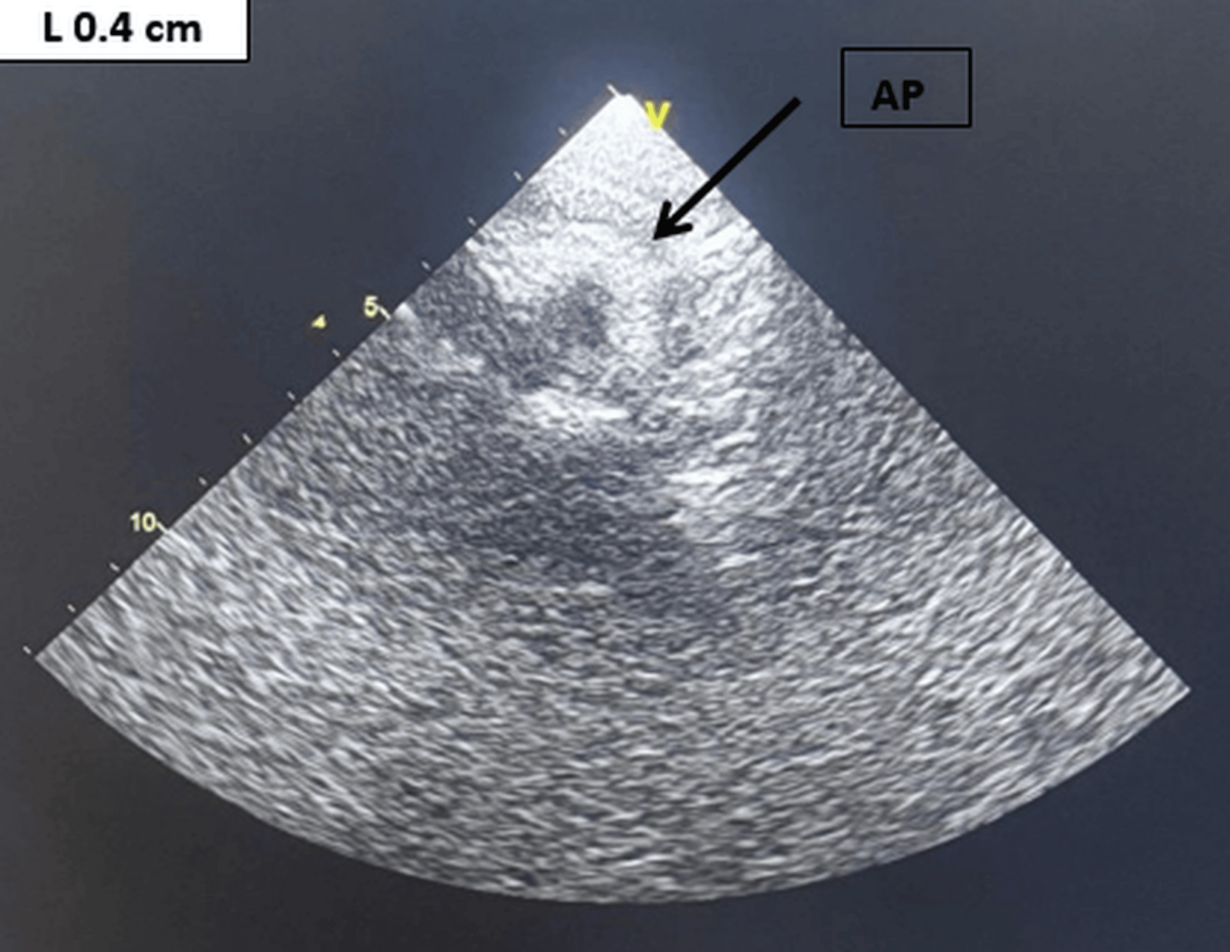
Two-dimensional echocardiographic image from the suprasternal window of the large atherosclerosis plaque in the aortic arch. The plaque thickness is 4 mm. AP is atherosclerosis plaque. The arrow indicates the atherosclerotic plaque in the aortic arch.

During cardiological follow-up, control echocardiography showed moderate regression of atheromatous lesions, with a reduction in aortic parietal thickening to 4 mm (Figure 5).

A six-monthly follow-up was instituted, including transaminase, CPK, and lipid profile determination, as well as regular cardiological monitoring. However, due to the high cardiovascular risk associated with this familial form of hypercholesterolemia, the possibility of foreign LDL apheresis was considered and discussed with the family.

## Discussion

Familial hypercholesterolemia (FH) is a rare genetic disorder of major clinical importance due to its significant impact on cardiovascular risk. It results mainly from mutations in the LDLR, APOB, and PCSK9 genes, leading to dysfunction of low-density lipoprotein (LDL) metabolism and excessive accumulation of LDL cholesterol in plasma [1]. These genetic alterations disrupt the function of the LDL receptor, compromising its role in the uptake and recycling of LDL particles, thus promoting the early development of atherosclerosis [2].

The heterozygous form of FH is relatively common, with an estimated prevalence of 1/250, while the homozygous form remains extremely rare (1/1,000,000). The latter typically presents in childhood with LDL cholesterol (LDL-C) concentrations often exceeding 500 mg/dL, exposing patients to a major cardiovascular risk requiring early and intensive management [3].

**Figure 6:**
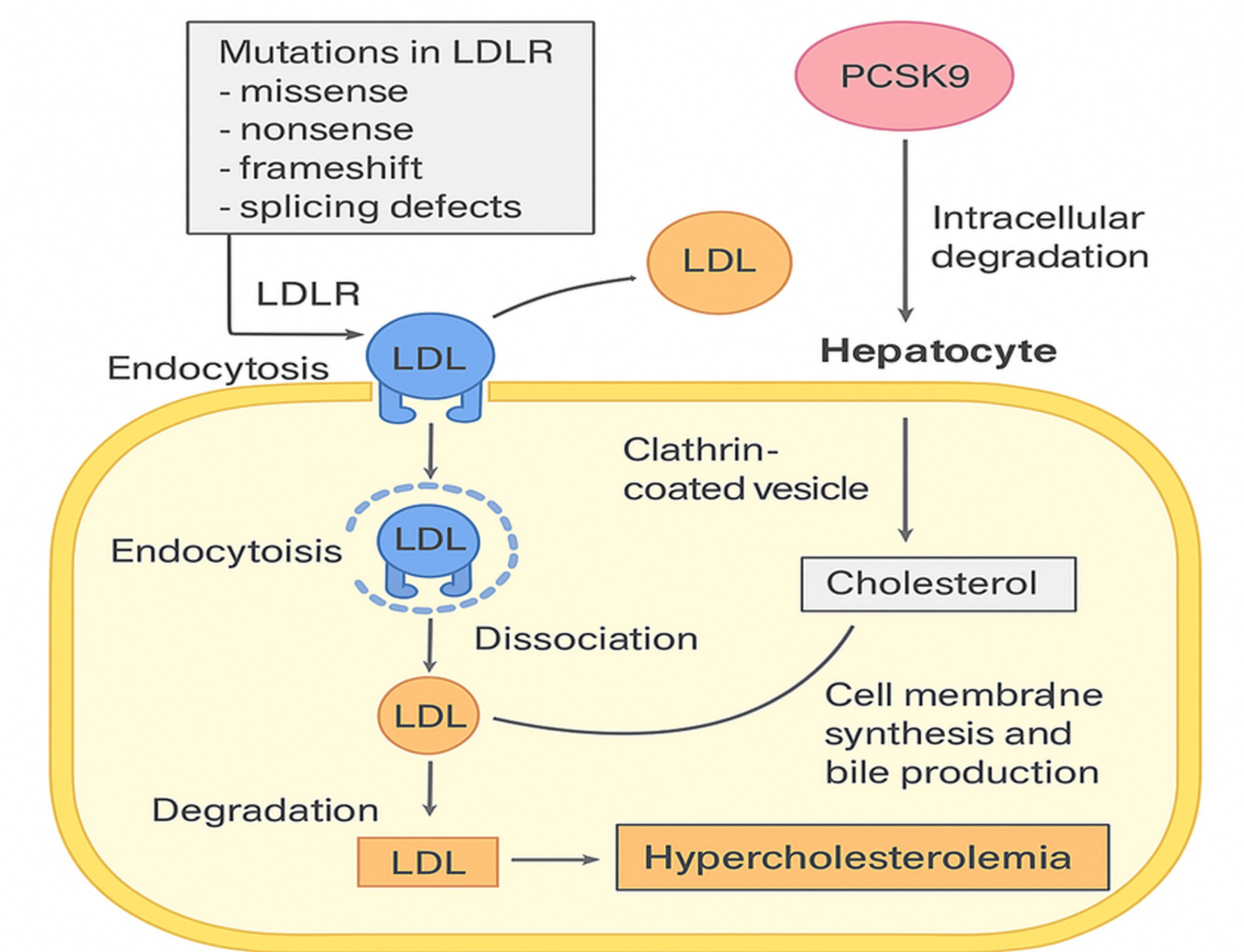
Mechanism of LDL metabolism: involvement of the LDL receptor Figure credits: Created by Professor Bensabbahia Dalal, with the aid of artificial intelligence to provide an accurate and reliable visual depiction of the data. LDL: low-density lipoprotein

The LDL receptor (LDLR) plays a central role in this pathology. This transmembrane protein, expressed on the surface of hepatocytes, facilitates the uptake and degradation of LDL particles via a multistep mechanism. It binds to apolipoprotein B-100 (ApoB-100) present on LDL particles, forming an LDLR-LDL complex. This complex is then internalized by endocytosis into clathrin-coated vesicles, allowing LDL particles to enter hepatocytes. Once inside the cells, LDL dissociates from the receptor, enabling its degradation and the liberated cholesterol to be used for various biological functions such as cell membrane synthesis and bile production [7]. Any disruption of this process, caused by mutations in the LDLR gene, seriously compromises the efficiency of this regulation. The most frequent mutations include missense, nonsense, frameshift, and splicing defects [4]. In addition, the PCSK9 protein plays a crucial role in negatively regulating the availability of LDL receptors on the surface of hepatocytes. It binds to LDL receptors and induces their intracellular degradation, thereby limiting their recycling and exacerbating hypercholesterolemia when overexpressed or hyperactive [8]. The mechanism is shown in Figure 6.

In our patient, a rare homozygous mutation in the LDLR gene was identified. This specific mutation (NM_000527.5:c.682G>C, p.Glu228Gln) is located in a critical functional region of the LDL receptor, leading to a significant loss of receptor activity resulting in severe hypercholesterolemia.

The Simon Broome classification is widely used for diagnosing familial hypercholesterolemia (FH), distinguishing between definite FH and possible FH based on biological, clinical, and genetic criteria. Definite FH is characterized by elevated total cholesterol and LDL cholesterol levels, associated with clinical signs such as xanthomas or the genetic confirmation of a pathogenic mutation. In contrast, possible FH is suggested by a family history of premature coronary artery disease or severe hypercholesterolemia, even in the absence of xanthomas or genetic confirmation [9]. The patient fulfills the definition of familial hypercholesterolemia category according to the Simon Broome criteria based on severe LDL-C elevation, tendon xanthomas, and family history. This classification aids in establishing a diagnostic probability and determining the likelihood of the disease, facilitating early management to reduce cardiovascular risk.

A study involving 264 children demonstrated an increased cardiovascular risk in those with a family history of premature coronary artery disease. These children showed elevated LDL cholesterol and apolipoprotein B levels, along with increased intima-media thickness - an early marker of atherosclerosis [10]. Although this specific assessment was not conducted in our patient, the presence of aortic atheroma in her case further underscores the high cardiovascular risk associated with familial hypercholesterolemia, highlighting the importance of early detection and timely intervention.

Management of homozygous familial hypercholesterolemia

In homozygous FH, children are at high risk of severe cardiovascular events due to the rapid progression of atherosclerosis. This early and aggressive disease course is often insufficiently controlled by conventional medical treatments, necessitating consideration of alternative therapies such as liver transplantation and LDL apheresis. These interventions can markedly reduce circulating LDL cholesterol levels, thereby decreasing cardiovascular risks [9].

The management of FH relies on a multimodal approach, combining dietary strategies, pharmacological treatment, and, in severe cases, specific interventions. A diet rich in plant-based fats and lean proteins, while limiting cholesterol intake, is essential for optimizing lipid control [10]. Pharmacological treatment, mainly based on statins, ezetimibe, and PCSK9 inhibitors, effectively lowers LDL cholesterol levels. In severe forms, LDL apheresis may be necessary to improve lipid control and reduce cardiovascular risk [11].

Importance of early diagnosis and genetic screening

Regular medical follow-up by a multidisciplinary team is essential to adjust treatment strategies and monitor vascular lesion progression. In this context, genetic screening plays a pivotal role in the early identification of at-risk individuals, enabling rapid diagnosis and targeted intervention. Although complex, genetic testing is crucial for optimizing therapeutic strategies and minimizing cardiovascular risks, allowing for preventive action before the onset of severe complications [12]. Diagnostic and therapeutic limitations were acknowledged, with practical implications suggested such as cascade family screening and inclusion in national registries or genetic follow-up programs.

## Conclusions

This case highlights the importance of careful clinical examination and targeted family history assessment in the early diagnosis of homozygous familial hypercholesterolemia. Genetic testing remains essential to confirm the diagnosis and guide therapeutic decisions, particularly in cases requiring intensive treatments. The clinical course demonstrated the regression of atheromatous plaques under treatment, although LDL levels remained persistently elevated, underscoring the complexity of disease management. Early multidisciplinary follow-up is crucial to prevent cardiovascular complications and improve long-term outcomes. This report also emphasizes the need to raise awareness among clinicians about the characteristic cutaneous and biological signs, to enable earlier recognition and intervention.
